# Soluble TREM-1 Serum Level can Early Predict Mortality of Patients with Sepsis, Severe Sepsis and Septic Shock

**DOI:** 10.1007/s00005-017-0499-x

**Published:** 2017-12-27

**Authors:** Monika Jedynak, Andrzej Siemiatkowski, Barbara Mroczko, Magdalena Groblewska, Robert Milewski, Maciej Szmitkowski

**Affiliations:** 1 Department of Anesthesiology and Intensive Therapy, Medical University of Bialystok, Bialystok, Poland; 2 Department of Neurodegeneration Diagnostics, Medical University of Bialystok, Bialystok, Poland; 3 Department of Biochemical Diagnostics, University Hospital of Bialystok, Bialystok, Poland; 4 Department of Statistics and Medical Informatics, Medical University of Bialystok, Bialystok, Poland; 5 Department of Biochemical Diagnostics, Medical University of Bialystok, Bialystok, Poland

**Keywords:** Sepsis, Prognosis, Inflammation, sTREM-1

## Abstract

Early prognostic prediction of sepsis is essential in adjusting therapeutic protocols to prevent deterioration and reduce mortality. We compared the predictive value of the serum concentration of the soluble triggering receptor expressed on myeloid cells 1 (sTREM-1) for 28-day mortality and for the development of severe sepsis or septic shock on the third day with the levels of interleukin (IL)-6, C-reactive protein (CRP) and procalcitonin (PCT). The study was conducted on 85 patients with sepsis. sTREM-1, CRP, PCT and IL-6 concentrations were measured upon study inclusion (day 0) and on days 1, 2, 3 and 5. APACHE II, SAPS II and SOFA scores were analyzed. The sTREM-1 levels (pg/ml) were higher in non-survivors than in survivors at admission (773 vs. 391, *p* < 0.001) and on days 1, 2, 3 and 5. In predicting the development of severe sepsis, the highest AUCs were found for PCT (0.744, 95% CI 0.638–0.85) and sTREM-1 (0.664, 95% CI 0.55–0.778); and in septic shock prediction, for PCT (0.766, 95% CI 0.665–0.867) and IL-6 (0.707, 95% CI 0.595–0.819). sTREM-1 positively correlated with APACHE II, SAPS II and SOFA scores. At inclusion, significant AUC for predicting 28-day mortality was 0.772 for the sTREM-1 (95% CI 0.672–0.871), 0.858 for APACHE II (95% CI 0.768–0.948), 0.847 for SAPS II (95% CI 0.733–0.96), 0.806 for SOFA score (95% CI 0.698–0.915). sTREM-1 can early predict the 28-day sepsis mortality, although its effectiveness is lower in comparison with clinical severity scores.

**Electronic supplementary material:**

The online version of this article (10.1007/s00005-017-0499-x) contains supplementary material, which is available to authorized users.

## Introduction

Despite considerable improvement in the pharmacological and supportive treatment of critical care patients, sepsis is one of the most frequent causes of hospital deaths, especially in intensive care units (ICUs) (Vincent et al. 2006). Although the mortality rate of patients with sepsis has been decreasing for the last 20 years, it still ranges between 29–33% for severe sepsis cases and can exceed 60% among septic shock cases (Kübler et al. 2015; Stevenson et al. 2014). Early diagnosis followed by prognostic sepsis assessment is crucial for providing effective treatment. Identifying patients at high risk of organ failure or shock is helpful when adjusting monitoring and treatment to prevent deterioration and to reduce mortality. Therefore, there is a need for a simple method that would facilitate early prognosis. Many studies are being conducted to identify a biomarker with high prognostic accuracy for sepsis, severe sepsis and septic shock.

Triggering receptor expressed on myeloid cells 1 (TREM-1) is an approximately 30 kDa transmembrane receptor protein composed of a signal peptide, an extracellular domain, a transmembrane domain and a short cytoplasmic domain of five amino acids (Pelham et al. 2014). The extracellular domain can be detected in the body fluids as soluble TREM-1 (sTREM-1), which functions as a decoy receptor. TREM-1 expression has been found on the surfaces of neutrophils, mature monocytes, macrophages and nonmyeloid cells, such as epithelial and endothelial cells (Pelham et al. 2014). Hypoxia, NF-κB, bacterial and fungal components can upregulate the expression of TREM-1, which is followed by the activation of monocytes and neutrophils, the production of proinflammatory cytokines, and degranulation and oxidative bursting by neutrophils. During an infection, the expression of membrane-bound TREM-1 is increased (Gibot and Cravoisy 2004; Gibot et al. 2005) and the level of sTREM-1 is elevated in plasma, bronchoalveolar lavage fluid and spinal fluid (Determann et al. 2006; Jiyong et al. 2009; Oudhuis et al. 2009). The usefulness of the serum sTREM-1 level for diagnosing infection has been analyzed (Brenner et al. 2017; Gibot et al. 2005; Kofoed et al. 2007; Schultz and Determann 2008; Wu et al. 2012) Similarly, several clinical studies were performed to assess the potential role of sTREM-1 in predicting mortality, and showed different prognostic effectiveness (Li et al. 2014, Su et al. 2013). Commonly used biomarkers such as C-reactive protein (CRP), procalcitonin (PCT) and interleukin (IL)-6 were not adequately effective for predicting the severity and mortality of sepsis (Adly et al. 2014; Palmiere and Augsburger 2014).

Considering these findings and the importance of the early assessment of the clinical course of sepsis, the prognostic value of sTREM-1 compared with CRP, PCT and IL-6 was analyzed in the present study. The aim of this study is to assess the effectiveness of sTREM-1 for predicting the development of severe sepsis or septic shock on the third day and 28-day mortality and among patients with sepsis, severe sepsis or septic shock.

## Materials and Methods

### Patients and Design

This prospective observational study was performed from March 2010 to February 2013 at a ten-bed mixed adult ICU at the Department of Anesthesiology and Intensive Therapy, Medical University of Bialystok. The Medical University Ethical Committee for research on humans and animals approved this study (no. R-I-002/272/2009). Written informed consent was obtained from all the subjects or their relatives. The admitted patients were evaluated within 12 h for systemic inflammatory response syndrome (SIRS) according to the criteria of the ACCP/SCCM Consensus Conference (Bone et al. 1992). Among 107 patients with SIRS, 85 were diagnosed with sepsis, severe sepsis or septic shock, according to the current guidelines (Dellinger et al. 2008; Levy et al. 2003). The final diagnosis was based on other clinical data, including diagnostic imaging and microbiological results. The exclusion criteria included the following: age younger than 18 years, disseminated malignant disease, pregnancy, AIDS or immunosuppressive treatment, life expectancy shorter than 24 h and lack of consent. The observation continued until death or discharge from the hospital, and 28-day mortality was the primary endpoint. The patients were treated according to the surviving sepsis guidelines.

### Measurements and Assays

The recorded data included age, gender, principal diagnosis, admission category, mortality, the lengths of mechanical ventilation and ICU stay, date of death, acute physiology and chronic health evaluation II (APACHE II) score, simplified acute physiology score (SAPS II), sequential organ failure assessment (SOFA) score, routine blood test and microbiological culture results, body temperature, CRP and PCT levels. An arterial blood was drawn and centrifuged at time of initial laboratory evaluation for sepsis (day 0), on days 1, 2, 3 and 5. Sera were stored at − 70 °C until analysis. Serum sTREM-1 and IL-6 levels were measured in duplicate in each sample using commercially available enzyme-linked immunosorbent assays (ELISAs; Human TREM-1 Quantikine ELISA Kit, Human IL-6 Quantikine ELISA Kit; R&D Systems, Minneapolis, MN, USA; lower detection limit 3.88 and 0.7 pg/ml, respectively), according to the manufacturer’s instructions. The PCT level (upper reference range 0.05 ng/ml in healthy subjects) was analyzed via an enzyme-linked fluorescent immunoassay for the quantitative measurement of PCT (VIDAS^®^ B·R·A·H·M·S PCT bioMérieux, France). The CRP level in the serum was determined via latex immunoassay using the Multigent CRP Vario Standard Method (Abbott Laboratories, Inc., USA and Abbott, Wiesbaden, Germany). The reportable range for the MULTIGENT CRP Vario measurement of CRP is 0.2–320 mg/l.

### Statistical Analysis

Because the data were not normally distributed, the results were presented as median and interquartile range. For comparison between the two groups, the nonparametric Mann–Whitney *U* test was used. The results for the qualitative variables are reported as percentages and were compared between groups using a Chi-square test. Correlations between different variables are presented as Spearman correlation coefficients with Bonferroni correction.

The prognostic values of different variables are expressed in terms of sensitivity, specificity and the areas under the receiver operating characteristic (ROC) curves and their 95% confidence interval (95% CI). Youden’s index was used to select the optimal cut-off values for the assessment of sensitivity, specificity and predictive values. To assess the independent prognostic value of sTREM-1 and other variables, we used a univariate logistic regression analysis that included the 28-day mortality as the outcome variable and sTREM-1 as the predictor. Thereafter, we built a multiple logistic regression model, adding routinely available tests and clinical scores to show the adjusted sTREM-1 odds ratios (OR).

Value *p* < 0.05 was considered statistically significant. The statistical analysis was performed using Statistica 10.0 for Windows.

## Results

### Patient Characteristics

A total of 85 adult ICU patients, including 54 men and 31 women, who met the criteria for SIRS and infection were prospectively included in this study; this set of patients was referred to as the systemic infection (SI) group. This group included 29 patients with sepsis, 32 with severe sepsis and 24 with septic shock; the median age was 68 years, ranging 55–75 years. For the SI group, the median length of ICU hospitalization was 16 days (range 5–30), and the median length of ventilation was 11 days (range 5–27). Among these 85 patients, 30 (35%) were admitted to the ICU during the first 24 h after a surgical procedure. There were 45 (53%) cases of pulmonary infection and 25 (29%) cases of abdominal infection. The most frequent cause of systemic infection was gram-negative bacteria (48.2%). The 28-day mortality rate was 16.5% for all the patients, 10% (*n* = 3) for patients with sepsis, 19% (*n* = 6) for patients with severe sepsis, and 21% (*n* = 5) for patients with septic shock. Three of the 85 patients died on the third day of observation. The mortality rate after 3 months of observation was 41%. The characteristics of the patients who died during the 28 days of treatment and those who survived are presented in Supplementary Table 1. The groups differed significantly in terms of age, serum pH, blood lactate level, APACHE II score, SAPS II and SOFA score at admission, the history of diabetes and arteriosclerosis.

### Serum Levels of sTREM-1, IL-6, CRP and PCT in Survivors and Non-survivors

**Fig. 1:**
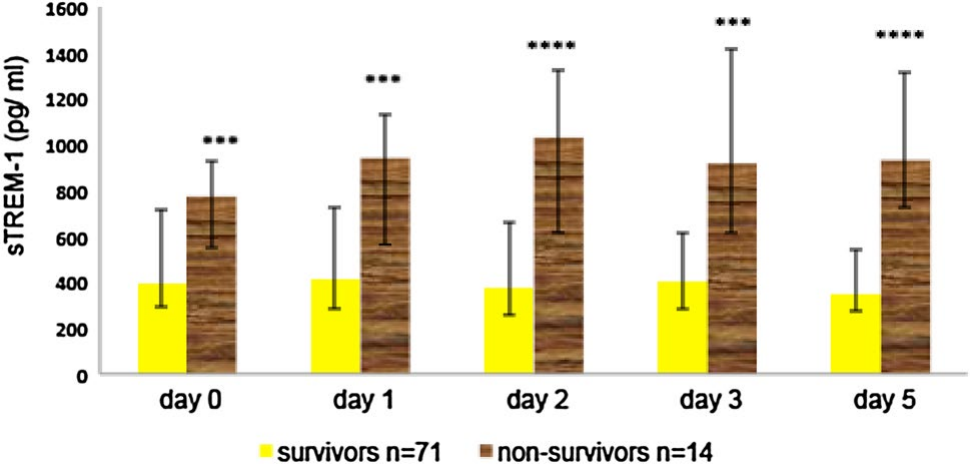
Serum levels of sTREM-1 in survivors (*n* = 71) and non-survivors (*n* = 14) with systemic infection, at study inclusion (day 0) and on days 1, 2, 3 and 5. The data are presented as median and interquartile range. ***p < 0.001; ****p < 0.0001

At admission (day 0), the median serum sTREM-1 level was significantly higher in the non-survivors than in the survivors (*p* < 0.001), and there were no differences in the CRP, PCT or IL-6 levels between these two groups (Table 1). Significantly higher sTREM-1 levels (pg/ml) were also found in the non-survivors than in the survivors on day 1 (943 vs. 411; *p* < 0.001), day 2 (1030 vs. 379; *p* < 0.0001), day 3 (918 vs. 408; *p* < 0.001) and day 5 (934 vs. 351; *p* < 0.0001), (Fig. 1). The CRP concentrations (mg/l) did not differ significantly between the survivors and the non-survivors for any day except day 5 (153 vs. 77; *p* < 0.05), (Supplementary Fig. 1). The median PCT level (ng/ml) in the non-survivors was higher than that in the survivors on day 1 (4.8 vs. 1.38; *p* < 0.01), day 2 (3.9 vs. 0.94; *p* < 0.0001), day 3 (6.3 vs. 0.6; *p* < 0.001) and day 5 (2.6 vs. 0.53; *p* < 0.001), (Supplementary Fig. 2). The median IL-6 concentrations (pg/ml) were higher in the non-survivors than in the survivors on day 2 (300 vs. 97; *p* < 0.05), day 3 (300 vs. 85; *p* < 0.05) and day 5 (300 vs. 78; *p* < 0.01) (Supplementary Fig. 3).

**Table 1:** Soluble concentrations of biomarkers on day 0 in survivors and non-survivors

Biomarker	Survivors *n* = 71 (83.5%)	Non-survivors *n* = 14 (16.5%)	*p* value
sTREM-1 (pg/ml)	391 (293–713)	773 (548–925)	< 0.001
CRP (mg/l)	148 (91–198)	105 (72–198)	0.387
PCT (ng/ml)	1.94 (0.82–7.1)	3.94 (2.2–14.9)	0.156
IL-6 (pg/ml)	220 (97–393)	300 (160–363)	0.458

The data are presented as median and interquartile range

*sTREM-1* soluble triggering receptor expressed on myeloid cells 1, *CRP* C-reactive protein, *PCT* procalcitonin

### sTREM-1, CRP, PCT and IL-6 at Inclusion for Predicting 28-Day Mortality

**Fig. 2:**
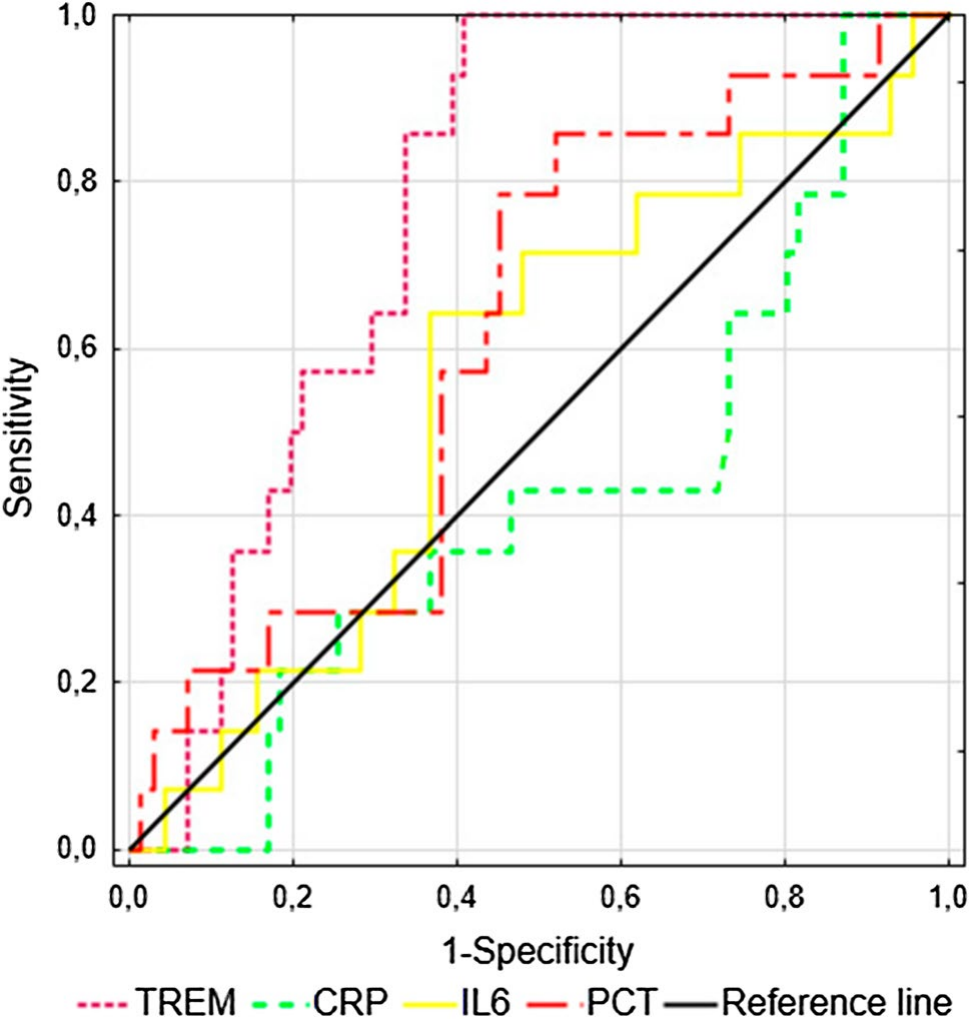
ROC curves for the prediction of the 28-day mortality in patients with sepsis, severe sepsis and septic shock (*n* = 85). The areas under the ROC curves (AUCs) for sTREM-1, CRP, PCT and IL-6 have been shown

The ROC curve analysis for predicting 28-day mortality at study inclusion showed an area under the curve (AUC) of 0.772 (95% CI 0.672–0.871) for sTREM-1, which displayed 86% sensitivity, 66% specificity, 33% positive predictive value, 96% negative predictive value and 69% accuracy at a cut-off value of 542 pg/ml. The AUCs for the CRP, PCT and IL-6 levels at admission did not present clinical value (Fig. 2).

### APACHE II, SAPS II and SOFA Scores at Inclusion for Predicting 28-Day Mortality

**Fig. 3:**
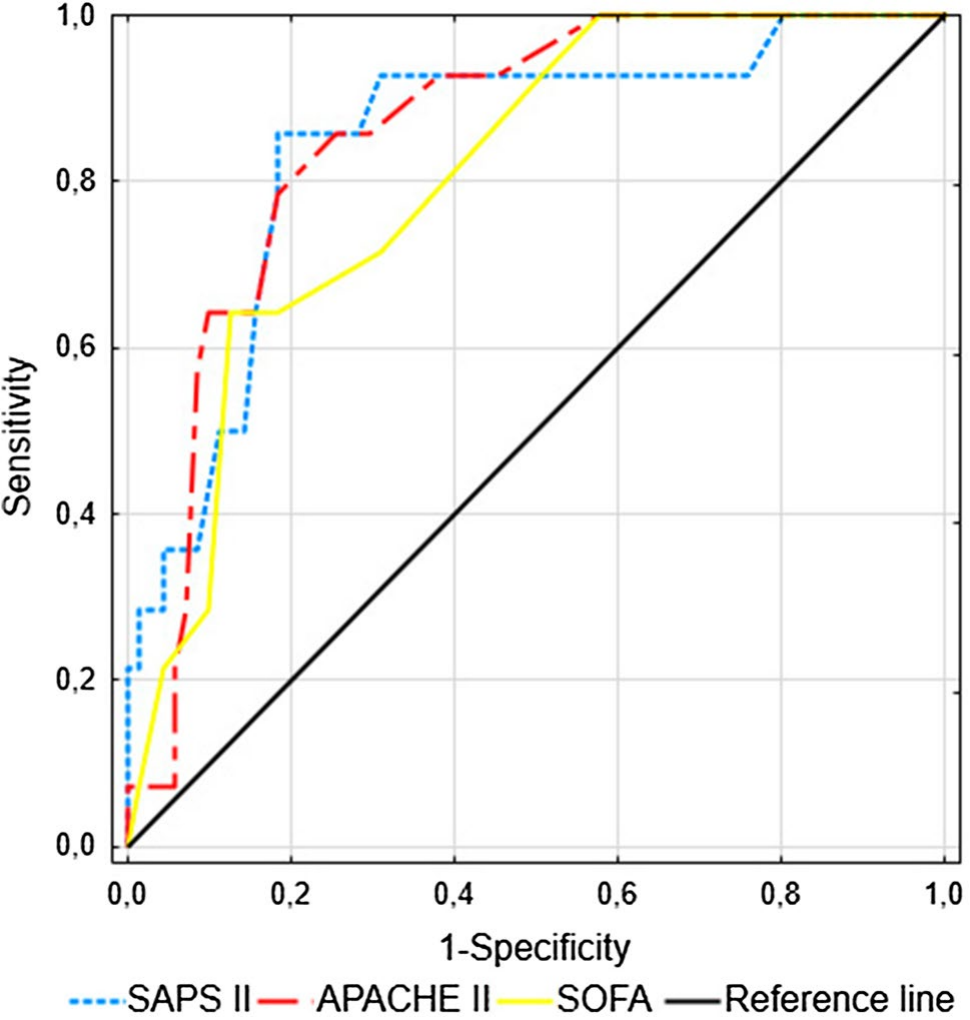
ROC curves for the prediction of the 28-day mortality in patients with sepsis, severe sepsis and septic shock *(n* = 85). The areas under the ROC curves (AUCs) for APACHE II score, SAPS II and SOFA score at study inclusion have been shown

A high prognostic value for predicting 28-day mortality was found for commonly used clinical scores, including the APACHE II (AUC: 0.858, 95% CI 0.768–0.948), SAPS II (AUC: 0.847, 95% CI 0.733–0.96) and SOFA scores (AUC: 0.806, 95% CI 0.698–0.915) (Fig. 3). The highest sensitivity (86%) was found for the APACHE II and SAPS II scores, which displayed specificities of 75 and 82%, respectively. The highest accuracy (84%) was shown for the SOFA score using a cut-off value of 12; this result was accompanied by 87% specificity.

### sTREM-1, CRP, PCT and IL-6 at Inclusion for Predicting Severe Sepsis or Septic Shock on the Third Day

**Fig. 4:**
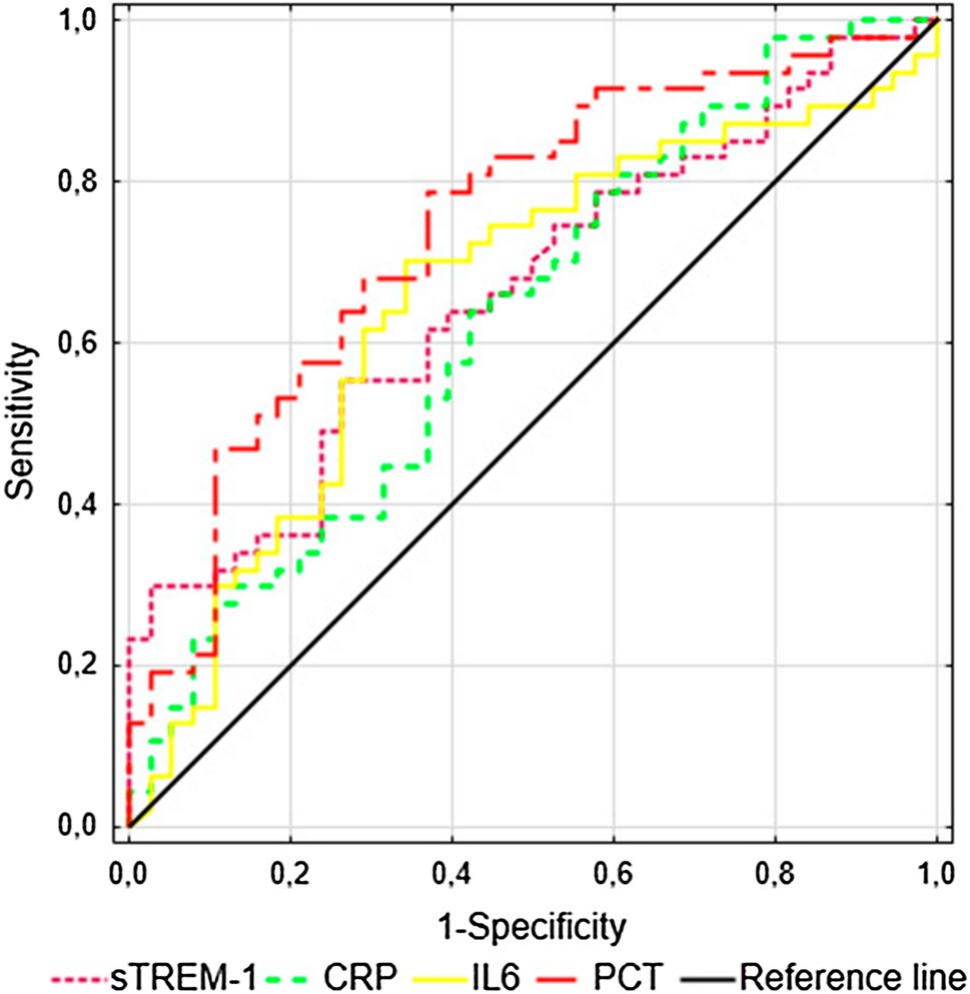
ROC curves for the prediction of severe sepsis on the third day in patients with sepsis, severe sepsis and septic shock (*n* = 85). The areas under the ROC curves (AUCs) for sTREM-1, CRP, PCT and IL-6 have been shown

**Fig. 5:**
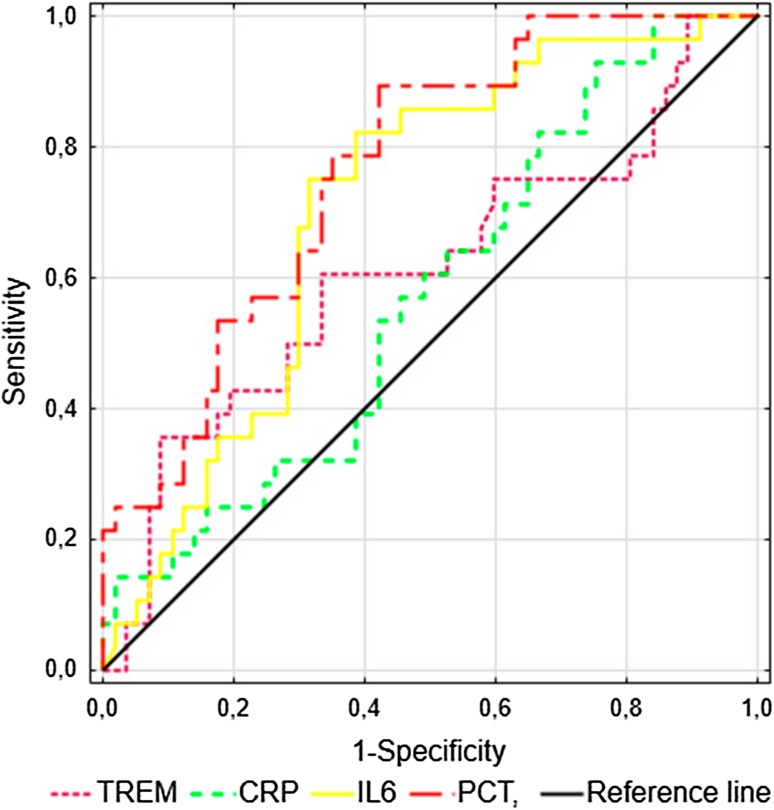
ROC curves for the prediction of septic shock on the third day in patients with sepsis, severe sepsis and septic shock (*n* = 85). The areas under the ROC curves (AUCs) for sTREM-1, CRP, PCT and IL-6 have been shown

To evaluate the potential value of the sTREM-1 level for predicting the deterioration of the clinical course, ROC curve analysis was performed. Of the 85 patients, 56 presented signs of severe sepsis or septic shock on the third day of treatment. Of those patients, six progressed from sepsis, six progressed from severe sepsis to septic shock, and the others did not improve. As a predictor of the persistence/development of severe sepsis on the third day of observation (*n* = 46), the highest AUC was shown for PCT, followed by sTREM-1, IL-6 and CRP (Fig. 4). The highest sensitivity (79%) and specificity (63%) were found for PCT using a cut-off value of 1.7 ng/ml (Supplementary Table 2). Similarly, IL-6 displayed 70% sensitivity and 66% specificity at a cut-off of 220 pg/ml. As a predictor of the persistence/development of septic shock on the third day of observation (*n* = 28), the highest AUCs were found for PCT (0.766, 95% CI 0.665–0.867) and IL-6 (0.707, 95% CI 0.595–0.819) (Fig. 5, Supplementary Table 2). Similarly, AUCs for the APACHE II, SAPS II and SOFA scores for predicting the development of severe sepsis or septic shock on the third day of treatment are presented in Supplementary Table 3.

### Correlation of sTREM-1 with Disease Severity and Inflammatory Mediators on the Day of Onset

On the day of onset, the soluble TREM-1 serum concentration correlated with clinical markers of severity, but not with the CRP level, the length of ICU stay, the length of artificial ventilation, age, heart rate, or mean arterial pressure (Table 2). The highest correlation coefficient (0.71; *p* < 0.00001) was found between the sTREM-1 level and the renal SOFA score, which is based on the serum creatinine level or urine output. To counteract the problem of multiple comparisons, the Bonferroni correction was used. Adjusted *p* values are presented in Table 2.

**Table 2:** Spearman’s rank correlations between the level of sTREM-1 on day 0 and clinical markers or inflammatory mediators

Clinical marker or inflammatory mediator	Serum sTREM-1 concentrations		
	*R*	*p* value	Adjusted *p* value^a^
APACHE II score	0.46	< 0.0001	< 0.01
SAPS II score	0.55	< 0.000001	< 0.0001
SOFA score	0.47	< 0.00001	< 0.001
Renal SOFA score	0.71	< 0.000001	< 0.0001
Lactate level	0.24	< 0.05	NS
White blood cell count	0.24	< 0.05	NS
Procalcitonin level	0.33	< 0.01	NS
IL-6 level	0.27	0.01	NS
CRP level	0.072	0.51	NS
Age (years)	0.187	0.087	NS

^a^Bonferroni correction

*APACHE II*, Acute Physiology and Chronic Health Evaluation II, *SAPS II* Simplified Acute Physiology Score II, *SOFA* sequential organ failure assessment, *NS* non-significant

### Added Prognostic Value of sTREM-1

A univariate analysis was performed to compare the OR of the variables collected on the day of sepsis onset for 28-day mortality. The sTREM-1 serum level, SAPS II, APACHE II score, SOFA score, renal SOFA score, age, steroid treatment, arteriosclerosis, diabetes, base access level and lactate level were associated with death (Table 3). However, after adjusting sTREM-1 to the APACHE II score, the SOFA score or the SAPS II score, the prognostic OR for sTREM-1 was not statistically significant (Table 4). Thus, determining the serum sTREM-1 level did not improve the prognostic value of commonly used clinical scores.

**Table 3:** Predictors of 28-day mortality in 85 patients with sepsis, severe sepsis and septic shock (univariate logistic regression)

Controlled variables	Odds ratio	95% CI	*p* value
sTREM-1	1.001	1.0–1.002	0.048
SAPS II	1.105	1.05–1.16	< 0.00001
APACHE II	1.246	1.106–1.403	< 0.00001
SOFA	1.495	1.176–1.899	0.001
Renal SOFA	1.917	1.23–2.99	0.004
Age (years)	1.075	1.013–1.141	0.017
Steroid treatment	8.03	2.306–27.97	0.001
Arteriosclerosis	4.92	1.026–23.62	0.046
Diabetes	3.827	1.045–14.01	0.043
Base access	0.902	0.819–0.992	0.033
Lactate concentration	1.44	1.059–1.96	0.02

*CI* confidence interval, *sTREM-1* soluble triggering receptor expressed on myeloid cells 1, *SAPS II* Simplified Acute Physiology Score II, *APACHE II* Acute Physiology and Chronic Health Evaluation II, *SOFA* sequential organ failure assessment

**Table 4:** Incremental value of sTREM-1 for predicting the 28-day mortality

Variables controlled	Adjusted OR for sTREM-1	*p* value
Crude OR	1.001 (1.0–1.002)	0.048
APACHE II	1.0004 (0.998–1.002)	0.629
SOFA	1.0007 (0.998–1.0024)	0.78
SAPS II	1.0003 (0.998–1.002)	0.655

*OR* odds ratio, *sTREM-1* soluble triggering receptor expressed on myeloid cells 1, *APACHE II* Acute Physiology and Chronic Health Evaluation II, *SOFA* sequential organ failure assessment, *SAPS II* Simplified Acute Physiology Score II

## Discussion

The present investigation showed that serum sTREM-1 measured within the first 24 h of ICU treatment represents a useful prognostic biomarker for patients with SI, including sepsis, severe sepsis and septic shock. Significantly higher sTREM-1 concentrations in non-survivors than in survivors were demonstrated upon ICU admission followed by stable elevation until day 5. An increased serum sTREM-1 level was associated with sepsis severity, as assessed according to the APACHE II, SAPS II and SOFA scores, and predicted the 28-day mortality with a sensitivity and specificity inferior to that of multifactorial scales. CRP, PCT and IL-6 admission levels were insufficiently sensitive or specific to differentiate septic patients with a high risk of death in the ICU. sTREM-1 was able to predict the development of severe sepsis, however, PCT was superior to sTREM-1, CRP and IL-6 in predicting severe sepsis and septic shock on the third day of treatment.

TREM-1 is a pattern-recognition receptor expressed on neutrophils and monocytes that is implicated in the development and amplification of the early inflammatory response to infection and injury. Because the blockade of TREM-1 protected mice against lipopolysaccharides-induced shock and sepsis was caused by *Escherichia coli*, this receptor was suggested as a potential therapeutic target (Bouchon et al. 2001). sTREM-1 is the soluble form of this receptor and is released into body fluids, when TREM-1 expression is upregulated (Gibot and Cravoisy 2004).

Gibot et al. (2005) and Gibot and Cravoisy (2004) showed a significant difference in monocytic TREM-1 expression between sepsis survivors and non-survivors on the third day of the disease and stable expression at a high level in the non-survivor group. Marioli et al. (2014) observed decreased TREM-1 gene expression in monocytes during the first 3 days of sepsis that was associated with an unfavorable outcome, but they also demonstrated a lack of correlation between the expression level and serum concentration of sTREM-1. Probably, as it was suggested, more complex mechanism regulates the concentration of the soluble form of this receptor. Concomitantly, the authors did not observe differences in sTREM-1 between survivors and non-survivors that may result from the dominance of patients with sepsis with low APACHE II score as well as the method of sTREM-1 measurement. The prognostic value of the soluble form of the receptor was also studied in 90 patients with sepsis, severe sepsis or septic shock by Giamarellos-Bourboulis et al. (2006). Significantly elevated serum sTREM-1 level was found at admission and throughout seven consecutive days in patients who died compared with those who survived. Similarly, Li et al. (2014) found significantly increased sTREM-1, PCT and IL-6 levels on day 0 of ICU treatment in 42 patients who died compared with 60 patients who survived. In contrary, PCT level differed on day 1 and IL-6 on day 2 between survivors and non-survivors in our research. This may result from the small size of non-survivors group, but even more from the different kinetics of biomarkers. Tomasiuk et al. (2014) was in agreement with our results and did not find a difference in the median PCT or IL-6 levels between survivors and non-survivors at admission.

According to our findings, the AUC for predicting the 28-day mortality was 0.772 for the sTREM-1 level, 0.858 for APACHE II score, 0.847 for SAPS II score and 0.806 for SOFA score. Li et al. (2014) reported very similar AUCs: 0.856 for the sTREM-1 level, 0.923 for the APACHE II score and 0.953 for the SOFA score. In contrast to our results, they found high AUCs for mortality prediction for PCT and IL-6 at admission; this discrepancy might have resulted from the larger sample size of non-survivors in their study or from the time of ICU admission. Corfield et al. (2014) presented the analysis of National Early Warning (NEW) score among patients admitted to the emergency department. The score is comprised of six physiological parameters including respiratory rate, oxygen saturation, temperature, systolic blood pressure, pulse, conscious level and one additional one such as oxygen supplementation. Points for each is summed to calculate the NEW score, ranging 0–20. The authors found the ability of the NEW score to predict in-hospital death within 30 days with an AUC of 0.70 (95% CI 0.67–0.74) and the association between increased score and high risk of adverse outcome of septic patients.

As independent prognostic variables, the APACHE II, SAPS II and SOFA scores were superior to sTREM-1 for predicting 28-day mortality in our study group. Incorporating the sTREM-1 serum level into clinical scoring systems such as the APACHE II, SAPS II and SOFA scores did not improve the prognostic value of these scores. Su et al. (2013) analyzed the prognostic value of sTREM-1, CRP, PCT, the SOFA score and other indicators in 100 patients with sepsis, severe sepsis or septic shock with a 28-day mortality rate of 43%. However, they observed significantly higher sTREM-1 and PCT concentrations in the non-survivor group, the ROC curve analysis showed that only the SOFA score can predict the 28-day mortality. A high value of sTREM-1 level with an AUC of 0.868 (95% CI 0.740–0.997) for predicting mortality was noted in blood culture-positive bacteremia patients (Su et al. 2012). Similarly, AUC of 0.68 (95% CI 0.58–0.78) for sTREM-1 for predicting hospital mortality was observed by Latour-Perez et al. (2010). The serum concentration of sTREM-1, but not IL-6 or IL-8, also predicted the ICU mortality and 28-day mortality in cancer patients with severe sepsis and septic shock (Ravetti et al. 2015).

For the first time, we demonstrated that PCT, sTREM-1, CRP and IL-6 were predictors of severe sepsis on the third day of SI; the PCT level had the highest AUC (0.744), with a sensitivity of 79% and a specificity of 63% at a cut-off value of 1.7 ng/ml. Similarly, PCT and IL-6, but not sTREM-1 or CRP, predicted the development of septic shock on the third day of SI treatment. Latour-Perez et al. (2010) confirmed our finding that sTREM-1 was positively correlated with the SAPS and SOFA scores at admission. We showed a strong correlation between the serum sTREM-1 level and the renal SOFA score (*R* = 0.71, *p* < 0.0001), that might result from impaired renal elimination of this factor. However, Su et al. (2011) showed higher urine concentrations of sTREM-1 in patients with sepsis-related acute kidney injury and suggested an association between the urine levels of sTREM-1 and renal injury. Thus, the correlation between the serum sTREM-1 level and renal failure needs further evaluation.

One strength of this study is that it involved a typical population of patients admitted to a mixed ICU and an observation throughout 5 days of treatment. However, our prospective observational study also had several limitations. First, patient recruitment was restricted to the periods in which the co-investigators worked as physicians. Second, because the non-survivor group was small in number, the sensitivity and specificity of some predictors might not have reached statistical significance. Third, not all the samples were collected before antibiotic therapy began, as that may have weakened results. We had no possibility to evaluate the cellular expression of TREM-1 in different immune cells together with the systemic response. Small size of the study, as well as the single center character are further limitations of present study.

Although several factors have been demonstrated to be associated with the high mortality of sepsis including age, liver cirrhosis, the degree of organ dysfunction and positive fluid balance, there remains a need for one simple parameter that enables health care professionals to classify patients according to the extent of intensive monitoring and treatment. The present study demonstrated the lack of efficacy of studied biomarkers as prognostic markers compared to the clinical scores. A systematic review (Minne et al. 2008) showed comparable and high performance of models based on SOFA scores with APACHE II/III or SAPS II in predicting mortality in ICU patients. Similarly, integration of metabolic and inflammatory mediators levels was found to serve as reliable prognostic tool for septic shock (Mickiewicz et al. 2015). Thus, the combination of number of clinical or biochemical parameters has still higher value than that of a single mediator, or we just did not find enough a strong biomarker to overpass the prognostic efficacy of multifactorial scales.

In conclusion, measuring the sTREM-1 level within 24 h of sepsis, severe sepsis or septic shock can be suitable for the prediction of 28-day mortality, albeit with lower accuracy than the commonly used APACHE II and SOFA scores. Incorporating the sTREM-1 serum level into clinical scoring systems does not improve the prognostic value of the mentioned scores. PCT was found to be the strongest predictor of severe sepsis and septic shock on the third day of treatment, and 1.8 ng/ml PCT can be used as a reference value in the clinical setting. We suggest that an elevated sTREM-1 level might predict sepsis-related acute kidney injury, but this requires further evaluation.

## Electronic supplementary material

Below is the link to the electronic supplementary material.

Supplementary material 1 (DOC 227 KB)

Supplementary material 2 (DOC 165 KB)

Supplementary material 3 (DOC 130 KB)

**Supplementary Fig. 1** Serum levels of C-reactive protein (CRP) in survivors (*n* = 71) and non-survivors (*n* = 14) with systemic infection, at study inclusion (day 0) and on days 1, 2, 3 and 5. The data are presented as median and interquartile range. *p < 0.05 (EPS 204 KB)

**Supplementary Fig. 2** Serum levels of procalcitonin (PCT) in survivors (n = 71) and non-survivors (n = 14) with systemic infection, at study inclusion (day 0) and on days 1, 2, 3 and 5. The data are presented as median and interquartile range. **p < 0.01; ***p < 0.001; ****p < 0.0001 (EPS 167 KB)

**Supplementary Fig. 3** Serum levels of interleukin (IL)-6 in survivors (*n* = 71) and non-survivors (*n* = 14) with systemic infection, at study inclusion (day 0) and on days 1, 2, 3 and 5. The data are presented as median and interquartile range. *p < 0.05; **p < 0.01 (EPS 186 KB)
