# Supplementary material for: Soluble TREM-1 Serum Level can Early Predict Mortality of Patients with Sepsis, Severe Sepsis and Septic Shock
Source: Arch Immunol Ther Exp (Warsz). 2017 Dec 27;66(4):299–306. doi: 10.1007/s00005-017-0499-x (PMC6061141; doi:10.1007/s00005-017-0499-x)
Supplement: Supplementary file 1 — Supplementary material 1 (DOC 227 KB) [file 5_2017_499_MOESM1_ESM.doc]

Soluble triggering receptor expressed on myeloid cells-1 serum level can early predict mortality of patients with sepsis, severe sepsis and septic shock.

Monika Jedynak, Andrzej Siemiątkowski, Barbara Mroczko, Magdalena Groblewska, Robert Milewski, Maciej Szmitkowski

**The affiliations and addresses of the corresponding author:**

Monika Jedynak MD, Department of Anesthesiology and Intensive Therapy, Medical University of Bialystok, M. Sklodowskiej-Curie 24a, 15-276 Bialystok Poland, monika.jedynak@umb.edu.pl

**Supplementary Table 1.** Clinical characteristics of the survivors and non-survivors of systemic infection (SI)

| **Characteristic** | **Survivors**  n = 71 (83.5%) | **Non-survivors**  n = 14 (16.5%) | **p value** |
| --- | --- | --- | --- |
| Age (years) | 64 (52-74) | 76.5 (72-78) | < 0.01 |
| Length of ICU stay (days) | 20 (12-30) | 6 (5-8) | < 0.0001 |
| Length of ventilation (days) | 14 (8-27) | 6 (5-8) | < 0.01 |
| Male/female (n) | 47/24 | 7/7 | 0.25 |
| Surgical admission (n, %) | 25 (35.2) | 5 (35.7) | 0.57 |
| ^a^SAPS II score* | 38 (33-45) | 56 (51-73) | < 0.0001 |
| ^b^APACHE II score* | 15 (12-20) | 25 (21-26) | < 0.0001 |
| ^c^SOFA score* | 9 (6-10) | 12 (9-13) | < 0.001 |
| Serum pH* | 7.41 (7.31-7.46) | 7.32 (7.29-7.41) | < 0.05 |
| Lactate level (mmol/l)* | 1.9 (1.2-2.6) | 2.7 (1.9-4.34) | < 0.05 |
| ^d^MAP (mmHg)* | 67 (60-73) | 66.5 (53-75) | 0.82 |
| Heart rate (beats per minute)* | 120 (100-130) | 120 (100-140) | 0.61 |
| Effective antibiotherapy (n, %) | 56 (78.9) | 5 (35.7) | 0.001 |
| ^e^CVVHD in the ICU (n, %) | 9 (12.7) | 4 (28.6) | 0.13 |
| Steroids in the ICU (n, %) | 13 (18.3) | 9 (64.3) | 0.001 |
| Arteriosclerosis (n, %) | 39 (55) | 12 (85.7) | < 0.05 |
| Diabetes (n, %) | 9 (12.7) | 5 (35.7) | < 0.05 |
| Microbiology | | | |
| Multiple pathogens (n, %) | 16 (22.5) | 2 (14.3) | 0.49 |
| Gram+ infection (n, %) | 21 (29.6) | 3 (21.43) | 0.54 |
| Gram– infection (n, %) | 32 (45.1) | 7 (50) | 0.74 |
| Fungal infection (n, %) | 9 (12.7) | 2 (14.3) | 0.87 |
| Infection site | | | |
| Pulmonary (n, %) | 37 (52.11) | 8 (54.12) | 0.73 |
| Abdominal (n, %) | 20 (28.2) | 5 (35.7) | 0.57 |
| Urinary tract (n, %) | 3 (4.23) | 0 (0) | 0.43 |
| Bloodstream (n, %) | 7 (9.9) | 1 (7.14) | 0.75 |
| Soft tissue (n, %) | 3 (4.23) | 0 (0) | 0.43 |

Quantitative data with a nonnormal distribution are presented as medians and interquartile ranges. Qualitative data are presented as n (%). ^a^ Simplified Acute Physiology Score II; ^b^ Acute Physiology and Chronic Health Evaluation II; ^c^ Sequential Organ Failure Assessment; ^d^ mean arterial pressure; ^e^ continuous venovenous hemodialysis; * values recorded on day 0.
