# Supplementary material for: Soluble TREM-1 Serum Level can Early Predict Mortality of Patients with Sepsis, Severe Sepsis and Septic Shock
Source: Arch Immunol Ther Exp (Warsz). 2017 Dec 27;66(4):299–306. doi: 10.1007/s00005-017-0499-x (PMC6061141; doi:10.1007/s00005-017-0499-x)
Supplement: Supplementary file 2 — Supplementary material 2 (DOC 165 KB) [file 5_2017_499_MOESM2_ESM.doc]

Soluble triggering receptor expressed on myeloid cells-1 serum level can early predict mortality of patients with sepsis, severe sepsis and septic shock.

Monika Jedynak, Andrzej Siemiątkowski, Barbara Mroczko, Magdalena Groblewska, Robert Milewski, Maciej Szmitkowski

**The affiliations and addresses of the corresponding author:**

Monika Jedynak MD, Department of Anesthesiology and Intensive Therapy, Medical University of Bialystok, M. Sklodowskiej-Curie 24a, 15-276 Bialystok Poland, monika.jedynak@umb.edu.pl

**Supplementary Table 2.** Value of inflammatory mediators on day 0 for predicting severe sepsis or septic shock on the 3^rd^ day of systemic infection in 85 patients with sepsis, severe sepsis and septic shock

|  | **^a^sTREM-1**  **(pg/ ml)** | | **C-reactive protein (mg/ l)** | | **Procalcitonin (ng/ ml)** | | **Interleukin-6 (pg/ ml)** | |
| --- | --- | --- | --- | --- | --- | --- | --- | --- |
|  | Severe sepsis | Septic shock | Severe sepsis | Septic shock | Severe sepsis | Septic shock | Severe sepsis | Septic shock |
| Cut-off value | 444 |  | 135 |  | 1.7 | 1.79 | 220 | 225.5 |
| Sensitivity (%) | 64 |  | 66 |  | 79 | 89 | 70 | 82 |
| Specificity (%) | 61 |  | 55 |  | 63 | 58 | 66 | 61 |
| Positive predictive value (%) | 67 |  | 65 |  | 73 | 51 | 72 | 51 |
| Negative predictive value (%) | 58 |  | 57 |  | 71 | 92 | 64 | 88 |
| ^b^AUC (95% of confidence interval) | 0.664  (0.55-0.778) | 0.61  (0.475-0.745) | 0.632  (0.512-0.751) | 0.573  (0.446-0.699) | 0.744  (0.638-0.85) | 0.766  (0.665-0.867) | 0.657  (0.538-0.776) | 0.707  0.595-0.819 |
| Accuracy (%) | 62 |  | 61 |  | 72 | 68 | 68 | 68 |

^a^ Soluble triggering receptor expressed on myeloid cells-1; ^b^ area under the receiver operating characteristic curve.
