# Supplementary material for: Soluble TREM-1 Serum Level can Early Predict Mortality of Patients with Sepsis, Severe Sepsis and Septic Shock
Source: Arch Immunol Ther Exp (Warsz). 2017 Dec 27;66(4):299–306. doi: 10.1007/s00005-017-0499-x (PMC6061141; doi:10.1007/s00005-017-0499-x)
Supplement: Supplementary file 3 — Supplementary material 3 (DOC 130 KB) [file 5_2017_499_MOESM3_ESM.doc]

Soluble triggering receptor expressed on myeloid cells-1 serum level can early predict mortality of patients with sepsis, severe sepsis and septic shock.

Monika Jedynak, Andrzej Siemiątkowski, Barbara Mroczko, Magdalena Groblewska, Robert Milewski, Maciej Szmitkowski

**The affiliations and addresses of the corresponding author:**

Monika Jedynak MD, Department of Anesthesiology and Intensive Therapy, Medical University of Bialystok, M. Sklodowskiej-Curie 24a, 15-276 Bialystok Poland, monika.jedynak@umb.edu.pl

**Supplementary Table 3.** Value of clinical scores on day 0 for predicting severe sepsis or septic shock on the 3^rd^ day of systemic infection in 85 patients with sepsis, severe sepsis and septic shock

|  | **^a^APACHE II** | | **^b^SOFA** | | **^c^SAPS II** | |
| --- | --- | --- | --- | --- | --- | --- |
|  | Severe sepsis | Septic shock | Severe sepsis | Septic shock | Severe sepsis | Septic shock |
| Cut-off value | 17 | 22 | 8 | 8 | 48 | 47 |
| Sensitivity, % | 66 | 54 | 81 | 93 | 45 | 64 |
| Specificity, % | 76 | 91 | 55 | 49 | 87 | 83 |
| Positive predictive value, % | 78 | 75 | 69 | 47 | 81 | 64 |
| Negative predictive value, % | 64 | 80 | 70 | 93 | 56 | 83 |
| ^d^AUC (95% of confidence interval) | 0.751  (0.646-0.855) | 0.792  (0.692-0.892) | 0.703  (0.59-0.816) | 0.75  (0.641-0.859) | 0.69  (0.578-0.802) | 0.748  (0.635-0.862) |
| Accuracy, % | 71 | 79 | 69 | 64 | 64 | 77 |

^a^ Acute Physiology and Chronic Health Evaluation II; ^b^ Sequential Organ Failure Assessment; ^c^ Simplified Acute Physiology Score II; ^d^ area under the receiver operating characteristic curve.
